# Validating Enteroid-Derived Monolayers from Murine Gut Organoids for Toxicological Testing of Inorganic Particles: Proof-of-Concept with Food-Grade Titanium Dioxide

**DOI:** 10.3390/ijms25052635

**Published:** 2024-02-23

**Authors:** Yann Malaisé, Eva Casale, Aurélie Pettes-Duler, Christel Cartier, Eric Gaultier, Natalia Martins Breyner, Eric Houdeau, Lauris Evariste, Bruno Lamas

**Affiliations:** Toxalim UMR1331 (Research Centre in Food Toxicology), Toulouse University, INRAE, ENVT, INP-Purpan, UPS, 31027 Toulouse, France

**Keywords:** intestinal organoids, toxicity testing, food toxicology, inorganic particles, food additive titanium dioxide

## Abstract

Human exposure to foodborne inorganic nanoparticles (NPs) is a growing concern. However, identifying potential hazards linked to NP ingestion often requires long-term exposure in animals. Owing these constraints, intestinal organoids are a promising alternative to in vivo experiments; as such, an in vitro approach should enable a rapid and reliable assessment of the effects of ingested chemicals on the gut. However, this remains to be validated for inorganic substances. In our study, a transcriptomic analysis and immunofluorescence staining were performed to compare the effects of food-grade TiO_2_ (*fg*-TiO_2_) on enteroid-derived monolayers (EDMs) from murine intestinal organoids to the known impacts of TiO_2_ on intestinal epithelium. After their ability to respond to a pro-inflammatory cytokine cocktail was validated, EDMs were exposed to 0, 0.1, 1, or 10 µg *fg*-TiO_2_/mL for 24 h. A dose-related increase of the *muc2*, *vilin 1*, and *chromogranin A* gene markers of cell differentiation was observed. In addition, *fg*-TiO_2_ induced apoptosis and dose-dependent genotoxicity, while a decreased expression of genes encoding for antimicrobial peptides, and of genes related to tight junction function, was observed. These results validated the use of EDMs as a reliable model for the toxicity testing of foodborne NPs likely to affect the intestinal barrier.

## 1. Introduction

Manufactured inorganic particles (metals and minerals) are abundant in products used in daily life (e.g., cosmetics, textiles, building materials), including their use in foodstuffs as food colouring or anti-caking additives [1,2], or as antimicrobial agents or oxygen scavengers in food packaging [2,3]. For food additives, due to their chronic ingestion with the diet, health agencies are constantly re-evaluating their potential health risks for humans [4,5]. This is the case for the food-grade (*fg*) titanium dioxide (TiO_2_, referred to as E171 in the European Union (EU)), which is one of the most produced worldwide food additives [6,7], used as a whitener and brightness agent in many food [8] and pharmaceutical products [9,10]. In addition, due to its mixed composition of micro- and nanoparticles (NPs), *fg*-TiO_2_ is also representative of manufactured nanomaterials that expose the general population to NPs through the diet [11]. Studies have reported a wide range of effects on gut barrier integrity when TiO_2_-NPs accumulate in the intestine, inhibiting the growth of epithelial cells [12], altering nutrient absorption [13,14], and causing epithelial permeability defects [15,16,17], as well as leading to increased reactive oxygen species (ROS) production [18,19] and proinflammatory signalling [15,16,20,21,22], both in vivo and in vitro [4,23,24]. Previous results have also shown the ability of foodborne TiO_2_ particles to cross both the small intestine and colon barrier [20,25,26] and to induce genotoxic effects [18,19,27,28,29] while promoting the development of precancerous colorectal lesions in the colon [20,30].

In EU, the precautionary principle led to public policies banning the use of the *fg*-TiO_2_ in 2022 [4,31] based on its capacity to induce oxidative stress [18] and genotoxicity [32,33], as well as its potential to cause developmental impacts when in utero exposure occurs via the mother’s diet [15,16,34,35,36]. However, the use of *fg*-TiO_2_ is still authorised outside Europe, and food safety agencies worldwide have asked for additional studies. This requires further in vitro models or time-consuming in vivo experiments on rodents. To date, with regard to the intestine as the first target organ, mono- or co-cultures of intestinal cell lines are not relevant enough in terms of self-organization (polarization and three-dimensional (3D) structure) and do not represent the variety of cell types and functions found in the gut epithelium, i.e., absorptive enterocytes, secreting Paneth cells, enteroendocrine and goblet (mucus-producing) cells, and chemosensory tuft cells [37]. All of these have to be present in vitro to mimic in vivo conditions. Recent technical advances in stem cells and three-dimensional cultures have allowed for the use of intestinal organoids that closely recapitulate the architecture and cellular composition of the intestinal epithelium [38,39,40]. These indeed represent a good alternative model to classical in vitro cultures, as well as in vivo experiments, according to the animal ethics principle of Replacement, Reduction, and Refinement. For example, gut organoids have been used in several studies for modelling diseases, such as inflammatory bowel disease, or for exploring the interactions between pathogens and the epithelium, as well as the mechanisms of action and transportation of drugs, among other applications [41,42,43]. As the closed 3D geometry of gut organoids prevents direct access to the apical region of the epithelium, these applications require technically challenging methods such as organoid microinjection, which limits the routine use of organoids. Alternatively, opened-up 3D gut organoids to obtain an enteroid-derived monolayer (EDM) model, which resembles the physiologic gut lining in terms of cell variety, have been used for functional tissue barrier assays [44,45,46]. However, the interest in the use of EDMs for the toxicological testing of inorganic substances in the intestine, from their potential impacts on cell proliferation to cell functions and responses has been poorly addressed.

In the current study, the aim was to compare EDM responses to *fg*-TiO_2_ with the previously reported toxicity data for this chemical to determine whether this in vitro model can be a reliable tool for assessing the effects of inorganic substances on the gut epithelium. Mice were used to prepare the EDMs in order to directly compare the effects with the in vivo data that were mostly obtained from this species. We first evaluated the cellular response of a murine EDM model to a common pro-inflammatory stimulus using a cytokine cocktail in order to validate the EDM’s ability to physiologically react to an environmental stimulus. Second, and consistent with most of the reported toxicity data in the literature, we showed that the integrity of the gut (EDM) barrier in terms of cell proliferation/differentiation/apoptosis, genotoxicity, epithelial innate (anti-microbial) defences, and tight junction (TJ) function was altered after exposure to *fg*-TiO_2_ for 24 h. Together, these results validated the use of EDM prepared from murine intestinal organoids as a reliable alternative to conventional in vivo experiments for screening the effects of inorganic food additives on the gut epithelium, including NPs.

## 2. Results

### 2.1. EDM Model Is Functional and Able to Respond to Inflammatory Stress

**Figure 1:**
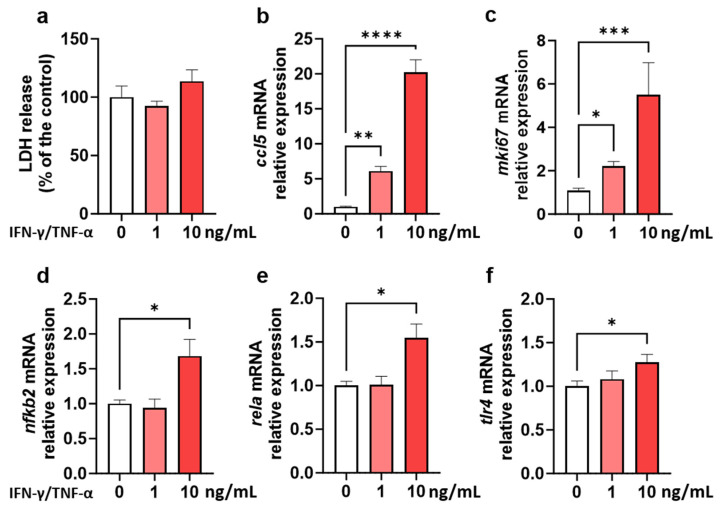
EDMs response to a pro-inflammatory cytokine cocktail. LDH release (**a**) and mRNA relative expressions of *ccl5* (**b**), *mki67* (**c**), *nfkb2* (**d**), *rela* (**e**), and *tlr4* (**f**) genes in EDMs exposed to 0, 1, and 10 ng/mL of IFN-γ/TNF-α cocktail for 24 h. Data are presented as mean ± SEM of three independent experiments, each with four to six EDMs per group. * *p* < 0.05, ** *p* < 0.01, *** *p* < 0.001, and **** *p* < 0.0001 using a Kruskal–Wallis test followed by a post hoc Dunn’s multiple comparison test.

Before assessing the impact of *fg*-TiO_2_ on EDMs, we first evaluated their ability to respond to an interferon (IFN)-γ/tumour necrosis factor (TNF)-α cocktail, used to mimic a pro-inflammatory stimulus. A dose–response study was carried out to ensure that the EDM model correctly responded to a range of biologically effective doses of an environmental stimulus without cytotoxicity (Figure 1). Compared to controls, no difference in lactate dehydrogenase (LDH) secretion was observed following IFN-γ/TNF-α exposure for 24 h, concluding on the absence of cytotoxicity for cytokine doses and the duration of treatment that were used (Figure 1a). The expression of genes known to be regulated by IFN-γ and/or TNF-α, such as chemokine (C-C motif) ligand 5 (ccl5), Toll-like receptor 4 (*tlr4*), ki67 (*mki67*), and nuclear factor-κB (*nfkb2*) and its p65 subunit (*rela*), were evaluated next. A dose-dependent up-regulation of *ccl5* and *mki67* was observed after 24 h exposure of EDMs to the IFN-γ/TNF-α cocktail (Figure 1b,c). At the highest dose of 10 ng/mL, these proinflammatory cytokines also induced an increase in the expression of *nfkb2*, *rela*, and *tlr4* genes compared to control EDMs (Figure 1d–f). These results highlighted that EDMs are functional and able to respond to inflammatory stresses during 24 h of culture, and that this model can be used to assess the direct impact of foodborne inorganic particles on the gut epithelial barrier, such as *fg*-TiO_2_.

### 2.2. fg-TiO_2_ Dose-Dependently Modulated the Expression of Genes Regulating Intestinal Barrier Function

**Figure 2:**
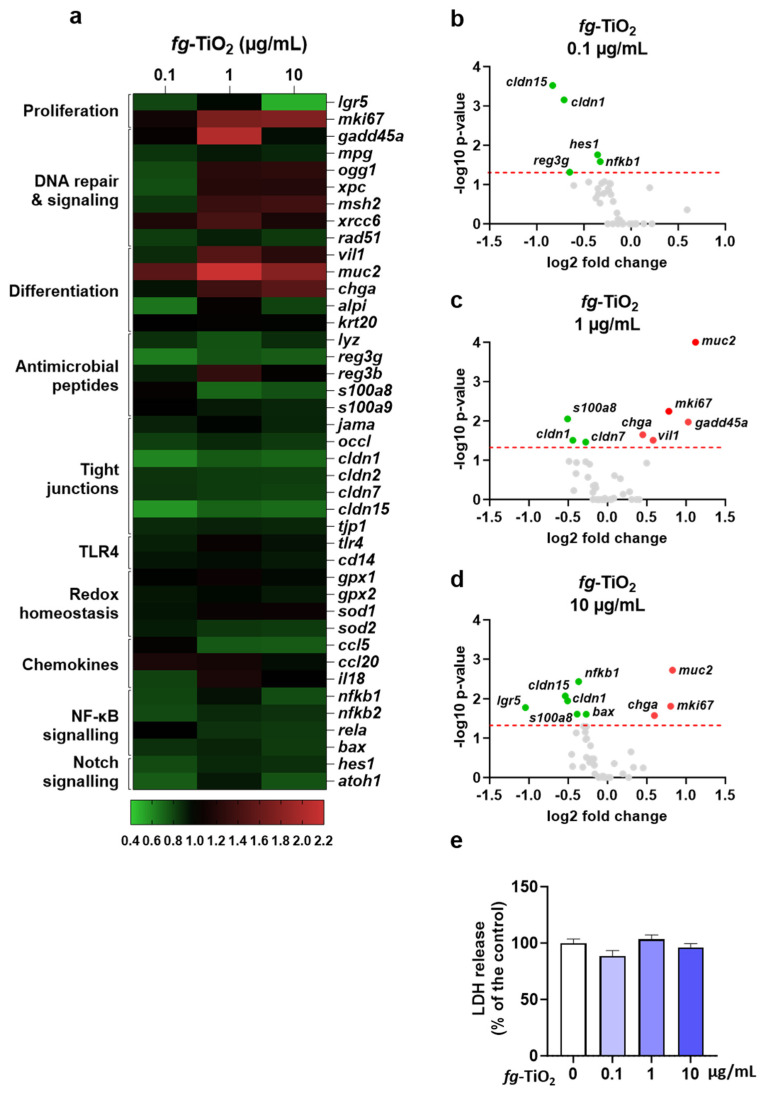
Differential expression of genes involved in the maintenance of the gut barrier after exposure of EDMs to *fg*-TiO_2_. (**a**) Heat map showing differential gene expression in EDMs exposed for 24 h to *fg*-TiO_2_ at 0.1, 1, and 10 µg/mL, compared to control. Red and green shadings represent higher and lower relative expression levels, respectively. (**b**–**d**) Volcano plot illustrating significantly different genes expressed in EDMs exposed for 24 h to *fg*-TiO_2_ at 0.1 (**b**), 1 (**c**), and 10 (**d**) µg/mL. Red and green dots represent significant (*p* < 0.05) higher and lower relative expression levels, respectively. Grey dots represent no significant change in relative expression levels. The red dashed line shows statistical significance threshold (adjusted *p*-values ≤ 0.05). (**e**) LDH release of EDMs exposed to *fg*-TiO_2_ at 0, 0.1, 1, and 10 µg/mL, where data are presented as the mean ± SEM of three independent experiments, each with four to six EDMs per group. Note that no significant LDH release was observed (Kruskal–Wallis test followed by post hoc Dunn’s multiple comparison test).

To investigate the effects of *fg*-TiO_2_ on gut barrier integrity and function, EDMs were exposed to the food additive at 0 (control), 0.1, 1, or 10 µg/mL for 24 h, and the expression of 41 genes participating in the maintenance of the gut barrier was evaluated (Figure 2a). A dose-dependent increase was observed in the number of genes that were differentially expressed (Figure 2b–d). At 0.1 µg/mL, 5 out of 41 genes were significantly down-regulated, while none of the others showed an altered expression compared to control EDMs (Figure 2b).

Next, the expression of eight and nine genes was significantly impacted following *fg*-TiO_2_ treatment at 1 and 10 µg/mL, respectively (Figure 2c,d). At these doses, the most differentially expressed and induced gene was the goblet cells marker mucin 2 (*muc2*), which encodes a mucin contributing to the mucus barrier of the intestine (Figure 2c,d). Furthermore, no difference in the level of LDH release was observed, regardless of the treatment dose, showing that all the EDMs remained viable, and that the observed transcriptomic effects consistently resulted only from their exposure to the food additive (Figure 2e). Overall, these results showed that a 24 h exposure of murine EDMs to *fg*-TiO_2_ induced a dose-dependent modulation of genes known as key players in the intestinal barrier function.

### 2.3. fg-TiO_2_ Altered Gene Expression Involved in the Secretory Cell Differentiation, Innate Defences, and Epithelial TJ Function of EDMs

**Figure 3:**
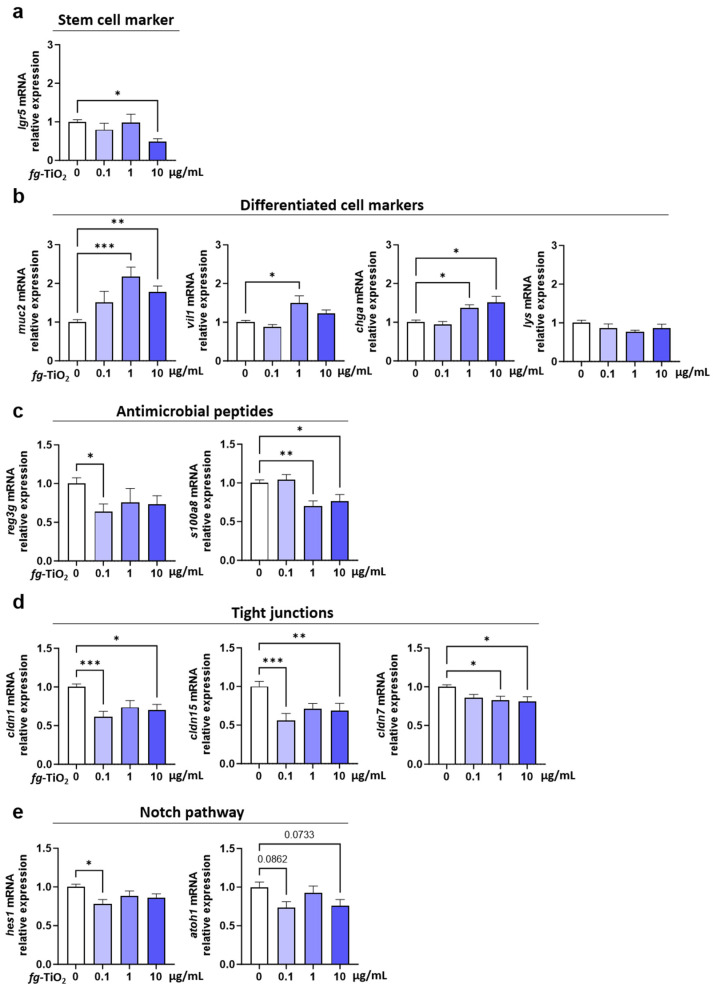
Effects of *fg*-TiO_2_ exposure on EDM gene markers of secretory cell, innate defences and epithelial TJ. Relative expression of genes from stem cells (**a**), differentiated cells (**b**), antimicrobial peptides (**c**), tight junctions (**d**), and notch pathway (**e**) in EDMs exposed to *fg*-TiO_2_ at 0, 0.1, 1, and 10 µg/mL for 24 h. Data are presented as the mean ± SEM of three independent experiments, each with four to six EDMs per group. * *p* < 0.05, ** *p* < 0.01, and *** *p* < 0.001 using one-way ANOVA followed by post hoc Dunnett’s multiple comparison test (**d**) or nonparametric Kruskal–Wallis test, followed by post hoc Dunn’s multiple comparison test (**a**–**c**,**e**).

A deeper analysis of the *fg*-TiO_2_-mediated transcriptomic effects revealed a decrease in the gene expression of the stem cell marker, leucine-rich repeat-containing G-protein coupled receptor 5 (lgr5), at the dose of 10 µg/mL (Figure 3a). In contrast, a dose-dependent increase in muc2 expression occurred, while a significant up-regulation of the enterocyte and enteroendocrine cell markers, Vilin (vil)1, and chromogranin (chg)A was observed at the highest doses of *fg*-TiO_2_ only (Figure 3b). On the other hand, no significant change was reported for the Paneth cell marker lysozyme (lyz), regardless of the dose (Figure 3b). Moreover, the gene expression of regenerating islet-derived 3γ (reg3γ), encoding a C-type lectin with bactericidal activity, and S100 calcium-binding protein A8 (s100a8), encoding another antimicrobial peptide in the gut and produced by epithelial cells or Paneth cells [47,48,49], was found to be decreased in *fg*-TiO_2_-treated EDMs (Figure 3c). For reg3γ mRNA, these effects were only significant at the dose of 0.1 µg/mL, while a significant down-regulation of s100a8 occurred at the two highest concentrations of the food additive. In addition, claudin (cldn)1, cldn7, and cldn15 mRNAs encoding tight-junction (TJ) proteins regulating epithelial (paracellular) permeability were decreased after *fg*-TiO_2_ treatment, with significance at 0.1 and 10 µg of *fg*-TiO_2_/mL for cldn1 and cldn15 and at the highest dose of the food additive for cldn7 expression (Figure 3d). In contrast, no change was noted for junctional adhesion molecule a (jama), occludin (occl), tight junction protein 1 (tjp1), and cldn2 genes (Figure S1). As claudin-1 also regulated the Notch pathway [50], which is involved in signalling cell fate and homeostasis in the gut, we investigated the gene expression of atoh1 (also named math-1) and its repressor hes1, which are both involved in Notch-signalling. EDM exposure to *fg*-TiO_2_ induced a reduction in hes1 expression at 0.1 µg/mL, while a slight but not significant drop in expression occurred for atoh1 (Figure 3e). Altogether, these results showed that the 24 h exposure of EDMs to *fg*-TiO_2_ down-regulated genes involved in innate defences and epithelial TJ permeability, while the gene expression of secretory cell markers was increased, suggesting a remodelling of the epithelium towards secretory lineages.

### 2.4. fg-TiO_2_ Increased Apoptosis and Induced Genotoxicity

**Figure 4:**
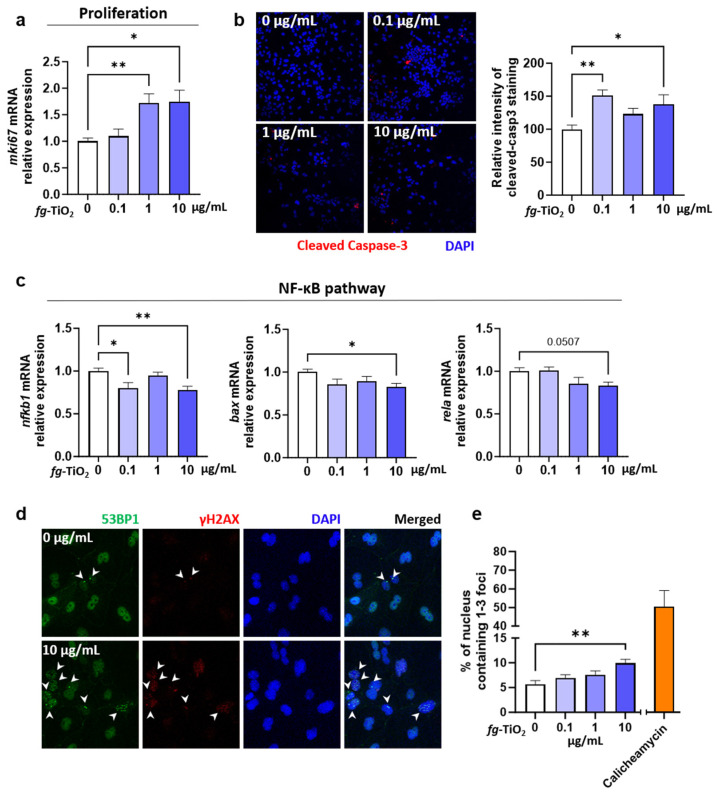
Effect of *fg*-TiO_2_ exposure on cell proliferation, apoptosis, and genotoxicity in EDMs exposed to *fg*-TiO_2_ at 0, 0.1, 1, and 10 µg/mL for 24 h. (**a**) Relative expression of *mki67* gene. (**b**) Immunofluorescence staining of cleaved caspase-3 (20× magnification) and the histogram showing the relative intensity staining compared to control. (**c**) Relative expressions of genes involved in NF-κB pathway. (**d**) Immunofluorescence staining of γH2AX and 53BP1 (20× magnification), white arrows pointing foci of γH2AX and 53BP1). (**e**) Percentage of nucleus containing 1–3 foci of γH2AX and 53BP1. EDMs exposed to calicheamycin γ-1 was used as positive control. Data are presented as the mean ± SEM of three independent experiments, each with four to six EDMs per group. * *p* < 0.05 and ** *p* < 0.01 using one-way ANOVA followed by post hoc Dunnett’s multiple comparison test (**e**) or nonparametric Kruskal–Wallis test, followed by post hoc Dunn’s multiple comparison test (**a**–**c**).

Rodent studies, as well as in vitro data using intestinal cell lines, have commonly reported that TiO_2_-NPs induce genotoxicity in the gut [18,20,27,28,29], which are known to impact cell proliferation and apoptosis. Therefore, the expression of markers involved in cell proliferation, apoptosis, and DNA damage was evaluated in EDMs treated with *fg*-TiO_2_. An increased mRNA expression of the proliferation marker *mki67* occurred at 1 and 10 µg/mL of the food additive (Figure 4a).

An immunofluorescence analysis showed a significant increase in the protein production of cleaved caspase-3 (a marker of apoptosis) when EDMs were treated with 0.1 and 10 µg/mL of *fg*-TiO_2_ compared to the control (Figure 4b). Accordingly, a down-regulation of the anti-apoptotic NF-κB pathway [51] occurred following the same treatments, characterized by the decreased mRNA expression of *nfkb1*, as well as of the subunits *rela* and *bax* (Figure 4c). Next, the genotoxic potential of *fg*-TiO_2_ was assessed by an immunofluorescence analysis of EDMs using antibodies directed against the tumour-suppressor p53-binding protein 1 (53BP1) and the phosphorylated serine 139 H2A histone family member X (γH2AX), two well-established DNA damage biomarkers [33]. In basal conditions, the 53BP1 staining appeared as a diffuse nuclear protein with the localization pattern of large nuclear speckles in cells (Figure 4d), named 53BP1 nuclear bodies [52]. In the presence of DNA double-strand breaks, 53BP1 is recruited to the damaged site and forms compact foci. The number of 53BP1 foci was systematically increased in EDMs exposed for 24 h to the highest dose of *fg*-TiO_2_ compared to unexposed controls (Figure 4d). We next evaluated the phosphorylation of γH2AX, which occurs at DNA double-strand breaks, also forming nuclei foci. As shown in Figure 4d, while only a few cells presented with γH2AX staining under control conditions, EDMs exposed to the *fg*-TiO_2_ exhibited accumulated γH2AX foci in the nuclei. A quantitative analysis of merged staining showed a dose-dependent increase in the percentage of nuclei, showing the colocalization of γH2AX and 53BP1 foci in EDMs exposed to *fg*-TiO_2_ (Figure 4e). When we focused on the DNA damage repair pathway genes (*mpg*, *ogg1*, *xpc*, *msh2*, *xrcc4*, and *rad51*), no significant change was reported, despite a tendency to increase at the two highest doses of the food additive (Figure S2a). Furthermore, for redox homeostasis genes (*gpx1*, *gpx2*, *sod1*, and *sod2*), no change was reported, regardless of the *fg*-TiO_2_ concentration (Figure S2b). Taken together, these results showed that the exposure of EDMs to *fg*-TiO_2_ induced DNA damage, together with increased apoptosis and the proliferation of epithelial cells.

## 3. Discussion

Given the increased evidence of the general population’s exposure to inorganic particles from various environmental sources (atmospheric ultrafine particles, livestock contamination by ground and feed, food additives, processing aids, personal care products, and pharmaceuticals) and the routes of exposure (airways, oral, and dermal), assessing the variety of toxicological impacts caused by these inorganic substances as part of the human exposome represents a complex challenge. For example, studies on food-related inorganic particles, such as certain food additives (mainly colouring and anti-caking agents), have highlighted the need for a permanent re-evaluation of their safety to human health, with a number of recent studies depicting new potential hazards, partly due to the presence of NPs in their composition [53,54,55]. This often requires long-term exposure carried out in vivo using rodent models, and cell lines of various organs including those that form barriers between the body and the environment, such as the intestine. Although animal studies continue to be central to answering these questions, the regulatory environment encourages alternative methods aiming to replace animal models [56]. However, in vitro experiments using intestinal cell lines lack cell diversity and specific cell functions to decipher the variety of effects of these food contaminants. In the current study, we showed that EDMs prepared from murine intestinal organoids, and exposed for 24 h at the apical luminal surface to an inorganic particle model commonly found in the human diet, namely the white pigment *fg*-TiO_2_, recapitulate most of the effects of this food additive on the gut found in long-term in vivo studies. These results validated the use of EDMs as a reliable alternative to in vivo experiments for the rapid toxicity testing of inorganic foodborne chemicals that are likely to affect the intestinal epithelium.

In order to validate the ability of this EDM model to physiologically and dose-dependently react to an environmental stimulus as a potential hazard signal, their response to a pro-inflammatory stimulus was first tested using a cocktail of IFN-γ/TNF-α cytokines. During pathogenic infection in the gut, innate and adaptive immune cells act together through IFN-γ and TNF-α secretion to enhance TLR4 mRNA and its protein expression by epithelial cells to induce pathogen recognition and innate immunity activation [57]. This TLR4 signalling can occur via MyD88-dependent or -independent pathways, involving the mitogen-activated protein kinase (MAPK) and the NF-κB pathway activations that lead to proinflammatory cytokine release [58,59,60]. The production of CCL5 by the intestinal epithelial cells was also regulated by TNF-α and IFN-γ, leading to inflammation maintenance and the migration of cells to the inflammatory site [61,62]. Finally, TNF-α-mediated epithelial proliferation in intestinal inflamed tissues was notably characterized by the increased number of Ki-67^+^ cells per crypt [63]. In our study, the EDM exposure to this cocktail consistently induced a dose-dependent up-regulation of *nfκb2* and its subunit *rela*, as well as of *ccl5*, *mki67*, and *tlr4*. These results confirmed that our EDM model is able to respond to inflammatory stresses and supported their use in evaluating the direct impact of *fg*-TiO_2_ on the gut epithelial barrier.

Following this validation step, the current study focused on a list of 41 genes of whose expression and protein products are involved in the regulation of the intestinal barrier [64,65,66,67]. In the presence of *fg*-TiO_2_, a dose-dependent modulation was observed, with five, eight, and nine of the forty-one genes whose expression significantly differed from the controls in EDMs exposed to 0.1, 1, and 10 µg/mL of this food additive, respectively. Among these genes, the expression of the stem cell marker *lgr5* was decreased at the highest dose of *fg*-TiO_2_. Zhang et al. showed a similar down-regulation of *lgr5* expression in mice and human gut organoids when exposed to 50 µg/mL of non-food TiO_2_-NPs (time of exposure not detailed), which is consistent with data on the mouse small intestine after two months’ exposure to the same dose of TiO_2_-NPs via drinking water [68]. This effect could explain previous in vivo studies showing modifications of intestinal epithelium morphology upon exposure to *fg*-TiO_2_, notably characterized by a reduction in the length of the crypts [69,70] which contain stem cells. Furthermore, the gene expression of markers commonly used to identify intestinal cells such as *vil1* (for enterocytes), *chga* (for enteroendocrine cells), *lyz* (for Paneth cells), and *muc2* (for goblet cells) [71,72,73] was determined to evaluate the effect of *fg*-TiO_2_ on the different cell types present in EDMs. An increased expression of *muc2*, *vil1*, and *chga* cell markers was observed in our study, while no change in the gene expression of the Paneth cell marker (*lyz*) occurred in *fg*-TiO_2_-exposed EDMs. The effects of TiO_2_ on the intestinal expression of *vil1*, *chga*, and *lyz* have been rarely or not evaluated, while studies exploring the impact of TiO_2_ on *muc2* expression or mucus production often reported contradictory results. Indeed, a co-culture of Caco-2 and HT29-MTX cells forming a regular mucus-secreting epithelium in vitro showed an increase in mucus secretion with no change in *muc2* gene expression after 21 days of exposure to 10, 50, or 100 µg/mL of *fg*-TiO_2_ [74]. Moreover, in vivo studies reported both decreased or increased *muc2* gene expression or goblet cell population after TiO_2_ exposure, depending on the time of exposure, the dose and the vehicle for treatment (i.e., gavage, drinking water or incorporation into food pellets) [27,69,70,75,76]. Our study showed that all epithelial cell types are present and reactive to an inorganic agent in our EDM model, and that 24 h exposure to *fg*-TiO_2_ altered the stem cell homeostasis (*lgr5*) in a dose-dependent manner while promoting the differentiation of secretory cells such as goblet (*muc2*) and enteroendocrine (*chga*) cells, suggesting a remodelling/restructuring of the intestinal epithelium mainly towards secretory lineages.

Given the importance of gut permeability in the systemic toxicity of chemicals, investigating the impact of xenobiotics on epithelial permeability is particularly relevant. Indeed, an alteration in this barrier after the ingestion of a xenobiotic can increase its passage into the bloodstream, as well as that of other environmental factors, such as pathogenic substances or opportunistic bacteria, with potential health consequences. Paracellular permeability along the gut epithelium is controlled by TJ protein complexes sealing cells between them, and is composed of transmembrane proteins of the claudin family, occludin, and junctional adhesion molecules, which are essential to the functioning of the physical gut barrier [77]. Of note, some discrepancies have been found in the literature regarding the ability of TiO_2_ to influence intestinal permeability, which could be related to whether data are obtained from the small or large intestine in vitro (i.e., by using Ussing chambers) or from the total gut in vivo (oral macromolecules), as well as the period of TiO_2_ exposure, i.e., whether this includes perinatal life or not. For instance, an increased in vivo intestinal permeability associated with a decreased expression in the jejunum of various genes related to intercellular junctions, such as *occl* and *cldn15*, was observed in male mice perinatally exposed to *fg*-TiO_2_ [15], while no permeability change occurred in this intestinal segment when exposure occurred in adulthood [25]. Another study also showed an increased permeability in the colon of mice perinatally exposed to *fg*-TiO2 [16], which was also not observed in adulthood [20]. In the ileum of adult mice, a down-regulation of *tjp1*, *tjp2*, *cldn2*, *cldn3*, and *occl* has been reported after a single oral gavage of TiO_2_-NPs at 12.5 mg/kg bw [26]. Similarly, a decreased mRNA expression of *tjp1* occurred in the colon of adult rats exposed to *fg*-TiO_2_ at 500 mg/kg bw/week for 10 weeks [78]. In our study, EDMs prepared with organoids obtained from small intestine stem cells of adult mice also exhibited a decreased expression of genes encoding TJ proteins, mainly *cldn1*, *cldn7*, and *cldn15*, after 24 h of exposure to *fg*-TiO_2_. However, expression of genes encoding for occludin and JAM-A were unaltered compared to controls. Both are key transmembrane proteins controlling intercellular spaces along the intestine [79,80]. This observation is consistent with the limited or no alteration of intestinal permeability when the adult gut is directly exposed to the food additive, in contrast to a perinatal treatment, which has consequences for the health of the offspring, making them more susceptible to food allergies or to developing colitis [15,16]. Finally, in addition to its role as a TJ protein sealing adjacent cells, claudin-1 also regulates gut homeostasis through the regulation of Notch signalling. Interestingly, using a villin-claudin-1 transgenic (Cl-1Tg) mouse model, the authors showed that the overexpression of claudin 1 led to Notch signalling activation, which, in turn, down-regulated *muc2* expression and inhibited goblet cell differentiation [50]. Therefore, one may hypothesize that the increase in *muc2* expression observed in *fg*-TiO_2_-treated EDMs could be partly due to the observed decrease in *cldn1* expression. This hypothesis is supported by the absence of Notch pathway activation in EDMs exposed to the food additive in the current study, which is consistent with another study showing no modulation of Notch signalling target gene *hes1* in mouse and human intestinal organoids exposed to 50 µg/mL of non-food TiO_2_-NPs [68].

In vitro, we also showed a decreased expression of the antimicrobial peptides *reg3γ* and *s100a8* genes after the *fg*-TiO_2_ treatment of EDMs. A down-regulation of *reg3γ* was reported in the colon of juvenile mice treated with TiO_2_ NPs at 10 and 40 mg/kg bw/day for 28 days [81]. The authors postulated that this effect may result from a direct alteration in the gut microbiota (namely gut dysbiosis) induced by sustained exposure to non-absorbed NPs in the gut lumen, affecting gut microbiota–host co-metabolites and leading to intestinal barrier damage [81]. However, in the in vitro model of EDMs that we used, i.e., in the absence of gut bacteria, the *fg*-TiO_2_-evoked decrease in *reg3γ* expression clearly suggested a microbiota-independent pathway for such regulation. This impact of the food additive could be linked to the increased expression of *muc2* observed here because, in vivo, mucin deficiency in Muc2 knockout mice enhanced the expression of *reg3γ* in the small intestine and colon [82]. Taken together, these in vivo data and our results using the EDM model suggested that, in addition to a direct impact of *fg*-TiO_2_ on intestinal bacteria [83,84,85], the food additive could also indirectly induce gut dysbiosis via a reduction in the secretion of antimicrobial peptides by epithelial cells, which is associated with an increase in mucus production in the intestine.

We further investigated whether the genotoxic potential of TiO_2_ that was previously reported in vivo and in vitro [18,20,27,28,29] is also observed in EDMs. Accordingly, the two markers of DNA double-stand breaks, γH2AX, and 53BP1 [86], accumulated and formed foci in EDMs exposed to *fg*-TiO_2_. Some studies have reported that TiO_2_-related genotoxicity mainly results from oxidative stress [19,87]. However, we did not observe changes in the expression of genes encoding for antioxidant enzymes, such as glutathione peroxidase 1 and 2 (*gpx1* and *gpx2*) and superoxide dismutase 1 and 2 (*sod1* and *sod2*). Consequently, it seems that some of the *fg*-TiO_2_-induced DNA damage in the intestine could not result from the induction of oxidative stress, at least at the transcriptomic level, which is concordant with the main conclusions in an in vitro study using a Caco-2 and HT29-MTX co-culture model [88]. Whatever the origin of the DNA lesions, DNA damage may interfere with the cell cycle and have consequences for cell proliferation and apoptosis [89,90]. Consistently, we report an increased expression of *mki67* and cleaved caspase 3 in EDMs exposed to *fg*-TiO_2_, suggesting a pro-proliferative and pro-apoptotic effect of the food additive. In human colon organoids, the significantly increased expression of apoptotic genes and proteins was also found after 48 h exposure to TiO_2_-NPs [91]. Furthermore, NF-κB pathway markers such as *nfkb1*, *rela*, and *bax* were found to be down-regulated in the current study. Interestingly, the NF-κB signalling pathway is activated in numerous cancers, leading to decreased apoptosis in malignant cells [92,93], and one may hypothesize from our study that the pro-apoptotic effect of the *fg*-TiO_2_ could be partly due to the inhibition of the NF-κB signalling pathway. Overall, these results support the genotoxic potential of *fg*-TiO_2_ using an EDM model, with DNA damage appearing independently of oxidative stress while leading to increased apoptosis, probably via inhibition of the NF-κB pathway.

In conclusion, the effects of *fg*-TiO_2_, described in our study using an EDM model for toxicological testing, are in concordance with the previously reported data on the intestinal effects of TiO_2_ (including NPs) when used as a food additive. Indeed, we showed that the integrity of the gut barrier, in terms of cell proliferation/differentiation, genotoxicity, innate defences, and epithelial TJs, is altered in murine EDMs exposed for 24 h to *fg*-TiO_2_. As our food-grade form of TiO_2_ (commercial E171 in EU) is representative of manufactured inorganic nanomaterials exposing the general population through the diet, this study suggests that EDMs, which recapitulate the complex cellular composition of the gut epithelium, could constitute a reliable tool for the rapid toxicological screening of inorganic foodborne chemicals. Indeed, these results support the capacity of EDMs to biologically respond to solid particles, and also the advantage of this model compared to 3D organoids in terms of the direct accessibility of the apical luminal surface of intestinal cells, which can expand and express conventional markers similar to in vivo situation. In this culture system, although the absence of other key players in the intestinal barrier function (i.e., immune cells, endothelial cells, and microbiota) limits the scope of the conclusion to the epithelium, the EDM model has the advantage of allowing for the direct effects of chemicals on the renewal and differentiation capacities of this physical barrier to be studied alone, i.e., without any other cellular compartment as an intermediary. The development of a co-culture with intestinal bacteria and other cell types would enable cell–cell and cell–bacteria interactions to be studied in order to reproduce physiological conditions, providing a powerful tool for toxicity studies.

## 4. Materials and Methods

### 4.1. Murine Intestinal Organoids

Five to eight-week-old male mice C57BL/6J (*n* = 3) were purchased from Janvier Labs (Le Genest-Saint-Isle, France) and housed for one week in the INRAE Toxalim animal facility (temperature: 22 ± 2 °C, relative humidity: 50 ± 20%, light/dark cycle: 12 h/12 h) with unlimited access to food (Mucedola 2018, Envigo, Milan, Italy) and water before being euthanised by cervical dislocation to collect intestinal crypts. To avoid any loss of intestinal crypts and organoids, all procedures described below were performed with Phosphate-Buffered Saline (PBS, Euromedex, Strasbourg, France) pre-wetted tubes and pipettes. Small intestine was collected, longitudinally opened, and cut into 3 fragments in a Petri dish containing cold PBS. After removing all faeces and intestinal content, the small intestine fragments were cut into 0.5 cm pieces, transferred to a 50 mL tube, washed by up and down pipetting, and then the supernatant was removed. This step was repeated 20–30 times. Small intestine pieces were then digested with Gentle Cell Dissociation (GCD) solution (STEMCELL Technologies, Grenoble, France) for 20 min at room temperature (RT) with gentle agitation. Supernatant was removed, and small intestine pieces were resuspended with cold PBS 0.1% Bovine Serum Albumin (BSA, Euromedex). After up and down pipetting, the supernatant was filtered with a 70 µm filter. This step was repeated to collect 7 fractions of cell suspension. All fractions were then centrifugated at 290× *g* for 5 min at 4 °C and supernatants were discarded. Pellets were washed with cold PBS 0.1% BSA and centrifugated again. All pellets were then resuspended in Dulbecco’s Modified Eagle Medium F12 (DMEM/F12) (STEMCELL Technologies) and the crypts were counted. Fractions containing at least 1500 crypts/mL were used for organoid culture. After a centrifugation at 290× *g* for 5 min at 4 °C, pellets were resuspended with cold Intesticult Organoid Growth Medium (OGM) mouse medium (STEMCELL Technologies) supplemented with 1% penicillin/streptomycin (Fischer Scientific, Strasbourg, France) (referred to here as complete mouse medium), and ice-cold Corning Matrigel (Fischer Scientific) at 1:1 ratio, and each crypt/Matrigel suspension was seeded in a pre-warmed 24-well plate at a density of 2500 crypts/50 µL. After incubation for 10 min at 37 °C, 5% CO_2_ for polymerization, organoids were cultured with complete mouse medium at 37 °C, 5% CO_2_. Media were replaced every 2–3 days, and organoids were passed every week. Briefly, organoids were mechanically broken by pipetting in GCD solution and centrifuged at 290× *g* for 5 min at 4 °C. Pellets were washed with DMEM/F12 and re-seeded with a dilution ratio 1:4 in 50 µL crypts/Matrigel suspensions and cultured with complete mouse medium at 37 °C, 5% CO_2_.

### 4.2. Preparation of Enteroid-Derived Monolayers (EDMs) and Treatments

After 4 passages, organoids were proceeded for EDM culture and treatments. To obtain EDMs, 3D organoids were first cultured in Intesticult OGM Human medium (STEMCELL Technologies) supplemented with 10 µM Y27632 (STEMCELL Technologies) and 1% penicillin/streptomycin (referred to here as complete Human medium) for 24 h. Organoids were then mechanically disrupted by pipetting in GIBCO TrypLExpress (Fischer Scientific) solution, collected, and incubated at 37 °C for 10 min for a total dissociation of the organoids. The reaction was stopped by adding a volume of DMEM/F12 and centrifuged at 290× *g* for 5 min at 4 °C. Pellets were resuspended with Human medium, and 2 × 10^5^ cells were seeded in a 2% Matrigel precoated 24 well-plate with or without coverslip, and cultured for 5 days at 37 °C 5% CO_2_ prior to treatment (Figure S3).

The *fg*-TiO_2_ was purchased as powder from the website of a French commercial supplier of food colouring agents, and was already characterized in previous studies as a representative E171 sample in the anatase crystal form that has been placed on the EU market [15,20,25,33,34,35]. Briefly, to obtain a stable dispersion of TiO_2_ particles, the *fg*-TiO_2_ stock suspension (10 mg/mL) was sonicated in an ice bath for 16 min at 30% amplitude with a VCX 750-230 V (Sonics&Materials, Newton, CT, USA), then stocked at 4 °C during 15 days maximum before use. In a first experiment, EDMs were exposed to a cytokine cocktail of IFN-γ (VWR, Paris, France) and TNF-α (VWR) at 1 or 10 ng/mL for 24 h to validate their physiologic and dose-dependent responses to a proinflammatory stimulus. EDMs treated with the vehicle (sterile water) were used as negative controls. In a second series, EDMs were exposed to *fg*-TiO_2_ at 0.1, 1, or 10 µg/mL for 24 h, then prepared for cytotoxicity assay, gene expression analysis, and immunofluorescence. As negative controls, EDMs were treated with the vehicle (sterile water). Calicheamicin γ1 (200 nM for 1 h) was used as a positive genotoxic control for γH2AX/53BP1 immunostaining.

### 4.3. Measurement of Lactate Dehydrogenase (LDH) Release

LDH release was measured using the CytoTox96 Non-Radioactive Cytotoxicity Assay kit (Promega, Lyon, France) according to the manufacturer’s instructions. Briefly, culture media of EDMs exposed to *fg*-TiO_2_ or cytokine cocktail for 24 h were collected, and the CytoTox reagent was added to each sample and incubated for 30 min at room temperature. The reaction was stopped using Stop Solution, and the absorbance at 490 nm was recorded with the Spark microplate reader (TECAN, Männedorf, Switzerland). LDH release was depicted as percent of the control.

### 4.4. Gene Expression Analysis

Total RNA was extracted from an EDM culture lysed in RLT buffer from the RNeasy Mini Kit (QIAGEN, Rennes, France), according to the manufacturer’s specification. A DNAse I digestion step was included in the purification protocol. The concentration and purity of total mRNA were measured using the N60 nanospectrophotometer (Implen, Munich, Germany). An amount of 400 ng of total RNA was used for cDNA synthesis using supermix iScript RT (BioRad, Marnes-la-Coquette, France), following the manufacturer’s protocol. cDNA was diluted 1:25 in nuclease-free water. Real-time Polymerase Chain Reaction (PCR) was performed using the Takyon ROX SYBR Mastermix blue dTTP (Eurogentec, Liège, Belgium) and specific primers (Table S1) on a Viaa7 Real-Time PCR System (Thermo Fisher Scientific, Waltham, MA, USA). Each assay was performed in duplicate, and the specificity of PCR products was verified using melting curve analysis. The relative expression level of the target genes was calculated using the ∆Ct method (2^−∆Ct^) and was normalized to the gene expression of the Ribosomal Protein L19 (RPL19). All expressions were relative to the untreated control (fold change).

### 4.5. Immunofluorescence

EDM cultures on coverslips were collected and fixed with 4% formalin for 30 min. All steps were performed at room temperature. EDMs were then permeabilised with PBS 0.2X Triton for 30 min and treated with NH_4_Cl for 30 min to remove residual formalin. EDMs were incubated in PBS 1% BSA for 1 h, followed by overnight incubation at 4 °C with a rabbit anti-mouse cleaved caspase-3 (Asp175) antibody (Cell Signaling Technology, Danvers, MA, USA), or a mix of mouse anti-vertebrate γH2AX (Ser139) (Sigma-Aldrich, Saint-Quentin-Fallavier, France) and rabbit anti-mouse 53BP1 (Novus Biologicals, Abingdon, UK) or rhodamine Phalloidin (Fischer Scientific). EDMs were then incubated for 2 h with a mix of Alexa Fluor 680 goat anti-mouse IgG (H+L) (Fischer Scientific) and Alexa Fluor 488 chicken anti-rabbit IgG (H+L) (Fischer Scientific) or with an Alexa Fluor Plus 680 Donkey anti-rabbit IgG (H+L) (Invitrogen, Waltham, MA USA, #A32802). EDMs were mounted in Prolong gold antifade mounting medium with 4′,6-diamino-2-phénylindole (DAPI) (Fischer Scientific) and examined under a Leica SP8 confocal microscope. Number of 53BP1 and γH2AX foci and cleaved caspase-3 fluorescence intensity were quantified with the ImageJ/Fiji software (version 1.54f).

### 4.6. Statistical Analysis

GraphPad Prism version 9.3.1 (GraphPad Software, Boston, MA, USA) was used for all analyses and preparation of graphs. The data are presented as the mean ± SEM from three independent experiments, each with 4 to 6 EDMs per group. Normal distribution was determined by a Kolmogorov–Smirnov test with Dallal–Wilkinson–Lillie correction. For datasets that failed normality tests, nonparametric tests were used. Multiple comparisons were evaluated statistically by one-way ANOVA, followed by Dunnett’s multiple comparisons tests or nonparametric Kruskal–Wallis tests, followed by Dunn’s multiple comparisons tests. Tests used are provided in each figure legend. Differences corresponding to *p* < 0.05 were considered significant.

## Data Availability

The data presented in this study are available upon request from the corresponding author.

## Supplementary Materials

The supporting information can be downloaded at: https://www.mdpi.com/article/10.3390/ijms25052635/s1.
