# Supplementary material for: Validating Enteroid-Derived Monolayers from Murine Gut Organoids for Toxicological Testing of Inorganic Particles: Proof-of-Concept with Food-Grade Titanium Dioxide
Source: Int J Mol Sci. 2024 Feb 23;25(5):2635. doi: 10.3390/ijms25052635 (PMC10932210; doi:10.3390/ijms25052635)
Supplement: Supplementary file 1 [file ijms-25-02635-s001.zip › ijms-2853792-supplementary.pdf]

Gene	Primers	Sequences	Gene	Primers	Sequences
lgr5	Forward	CGTAGGCAACCCTTCTCTTATC	*cldn1*	Forward	GCTGTCATTGGGGGCATAATA
	Reverse	GCACCATTCAAAGTCAGTGTTC		Reverse	GGGGTCAAGGGGTCATAGAAT
mki67	Forward	GAGGAGAAACGCCAACCAAGAG	*cldn2*	Forward	GGGCTGTTAGGCACATCCAT
	Reverse	TTTGTCCTCGGTGGCGTTATCC		Reverse	GTCGCACACTCCATCCAGAG
gadd45a	Forward	TGCGAGAACGACATCAACAT	*cldn7*	Forward	AATGTACGACTCGGTGCTCG
	Reverse	TCCCGGCAAAAACAAATAAG		Reverse	GTGTGCACTTCATGCCCATC
mpg	Forward	TGCATTTCTGGGACAGGTTCTT	*cldn15*	Forward	CGTGGGCAACATGGATCTCT
	Reverse	GAGTTCTGTTCCATCAGCGAGT		Reverse	CCACGAGATAGCCACCATCC
ogg1	Forward	TGAGCTGCGTCTGGACTTGGTT	*tjp1*	Forward	GCGCGGAGAGAGACAAGATG
	Reverse	CTCCGTCTGAGTCAGTGTCCAT		Reverse	CTGTGAAGCGTCACTGTGTG
xpc	Forward	GGTATTGTCGTGGAGAAGCAGTC	*tlr4*	Forward	AGCTTCTCCAATTTTTCAGAACTTC
	Reverse	CACGGTTAGAGAAGCCTTTCACC		Reverse	TGAGAGGTGGTGTAAGCCATGC
msh2	Forward	GAACAAAGGCGAGTATGAAGAGG	*cd14*	Forward	TTGAACCTCCGCAACGTGTCGT
	Reverse	GCGTCTAAGTGAGCCAGCACAT		Reverse	CGCAGGAAAAGTTGAGCGAGTG
xrcc6	Forward	GCAGTCTACTCCTGCCTAGTGA	*gpx1*	Forward	CGCTCTTTACCTTCCTGCGGAA
	Reverse	ACCTGGCTCATCAAACCGCTTC		Reverse	AGTTCCAGGCAATGTCGTTGCG
rad51	Forward	AGCAGTAGCTGAGAGATACGGT	*gpx2*	Forward	GAGGAACAACTACCCGGGACTA
	Reverse	CCCTCGCGCATATGCTACATTA		Reverse	ACCCCCAGGTCGGACATACT
vil1	Forward	CTCAAGACTCCGTCCTGCTG	*sod1*	Forward	GGTGAACCAGTTGTGTTGTCAGG
	Reverse	CCACTTGTTTCTCCGTCCGA		Reverse	ATGAGGTCCTGCACTGGTACAG
muc2	Forward	CTACCATTACCACCACTAC	*sod2*	Forward	TAACGCGCAGATCATGCAGCTG
	Reverse	GTCTCTCGATCACCACCATTT		Reverse	AGGCTGAAGAGCGACCTGAGTT
chgA	Forward	CAGGGACACTATGGAGAAGAGA	*ccl20*	Forward	GTGGGTTTCACAAGACAGATGGC
	Reverse	CTCTTGGTTAGGCTCTGGAAAG		Reverse	CCAGTTCTGCTTTGGATCAGCG
alpi	Forward	CCAGCAGTAACTCACCTCATGG	*ccl5*	Forward	CCTGCTGCTTTGCCTACCTCTC
	Reverse	GAAGCCTTGTGGATTCCTGCTG		Reverse	ACACACTTGGCGGTTCCTTCGA
krt20	Forward	GGATTCGAGGTTCAAGTCACGG	*il18*	Forward	GACAGCCTGTGTTCGAGGATATG
	Reverse	TCTAGGTTGCGCTCCAGAGACT		Reverse	TGTTCTTACAGGAGAGGGTAGAC
lyz	Forward	GTGCCTGTCCTGATCTTTCT	*nfkb1*	Forward	GCTGCCAAAGAAGGACACGACA
	Reverse	GATTTGCTCCTGTGGTTATTGG		Reverse	GGCAGGCTATTGCTCATCACAG
reg3g	Forward	CGTGCCTATGGCTCCTATTGCT	*nfkb2*	Forward	TGCTGATGGCACAGGACGAGAA
	Reverse	TTCAGCGCCACTGAGCACAGAC		Reverse	GTTGATGACGCCGAGGTACTGA
reg3b	Forward	ATGCTGCTCTCCTGCCTGATG	*rela*	Forward	TCCTGTTCGAGTCTCCATGCAG
	Reverse	CTAATGCGTGCGGAGGGTATATTC		Reverse	GGTCTCATAGGTCCTTTTGCGC
jama	Forward	CGCGTCGGGATTGTAACTGT	*bax*	Forward	AGGATGCGTCCACCAAGAAGCT
	Reverse	CACCGAACCCTTGCCTTGTA		Reverse	TCCGTGTCCACGTCAGCAATCA
s100a8	Forward	TCAAGACATCGTTTGAAAGGAAATC	*hes1*	Forward	TGAAGCACCTCCGGAACCT
	Reverse	GGTAGACATCAAATGAGGTTGCTC		Reverse	CAACACGCTCGGGTCTGTG
s100a9	Forward	AAAGGCTGTGGGAAGTAATTAAGAG	*atoh1*	Forward	AACTGTCCCTCCTGGATAGCA
	Reverse	GCCATTGAGTAAGCCATTCCC		Reverse	CCCTGCAAAGTGGGAGTCAG
occl	Forward	GTGAGCACCTTGGGATTCCG	*rpl19*	Forward	GAAGGTCAAAGGGAATGTGTTCA
	Reverse	TTCAAAAGGCCTCACGGACA		Reverse	CCTTGTCTGCCTTCAGCTTGT

Table S1. List of the primers used for the q-PCR.

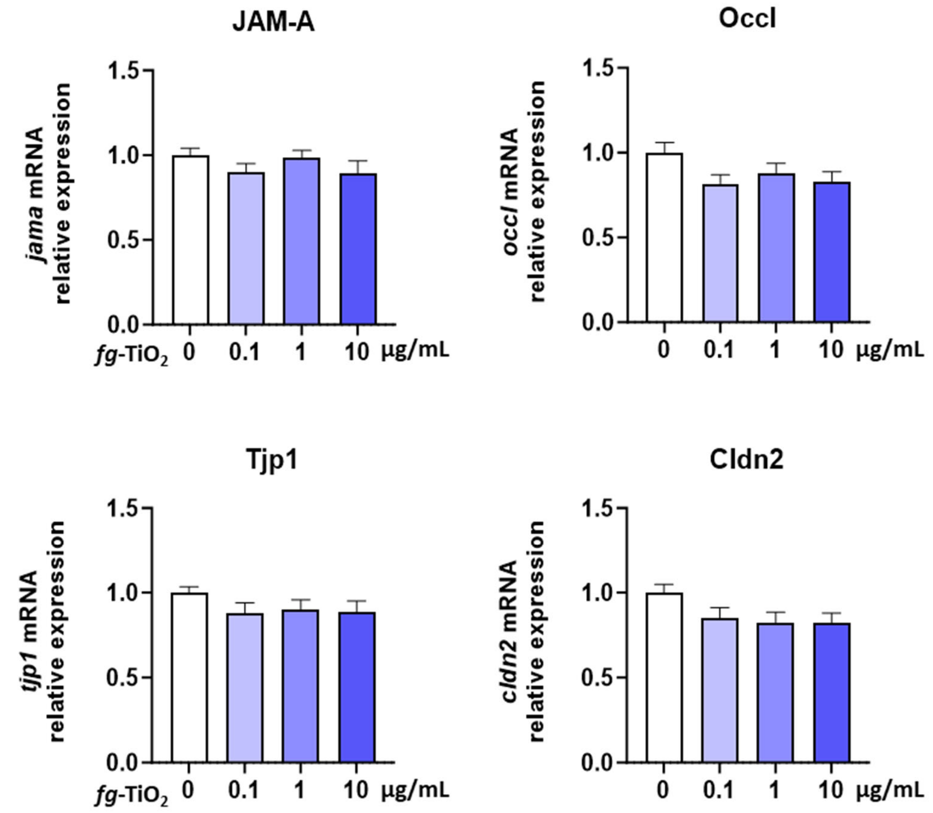

Figure S1. Relative expression of tight junction genes in EDMs exposed to *fg*-TiO_2. Relative expressions of *jama*, *occl*, *tjp1* and *cldn2* in EDMs exposed to *fg*-TiO_2 at 0.1, 1 and 10 µg/ml for 24h. Data are presented as the mean ± SEM of three independent experiments, each with 4 to 6 EDMs per group. Note that no significant difference was observed (one-way ANOVA followed by post hoc Dunnett's multiple comparison test).

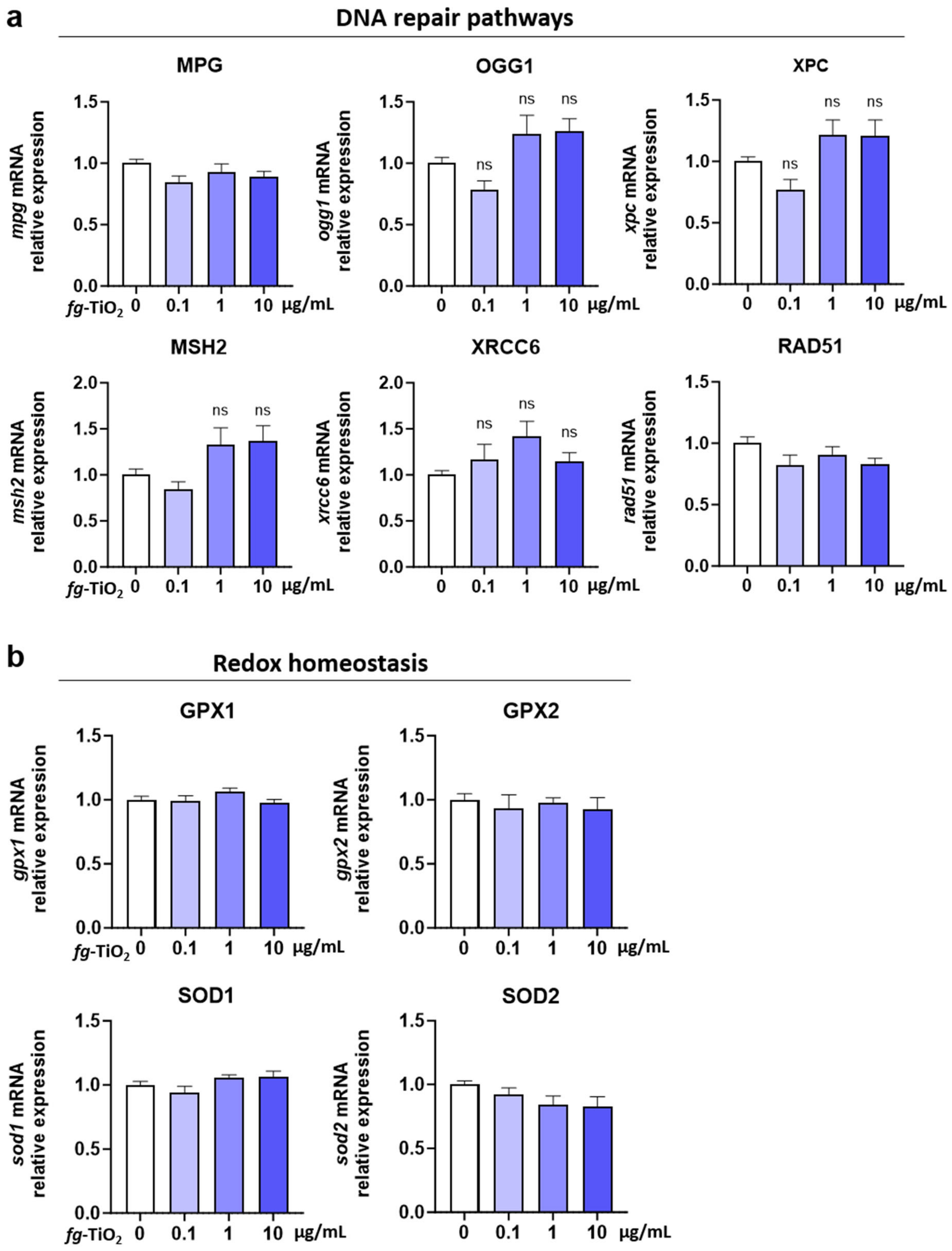

Figure S2. Relative expression of genes involved in DNA repair pathway and redox homeostasis in EDMs exposed to *fg*-TiO_2. Relative expressions of genes involved in DNA repair pathways **(a)** and in redox homeostasis **(b)** in EDMs exposed to *fg*-TiO_2 at 0.1, 1 and 10 µg/ml for 24h. Data are presented as the mean ± SEM of three independent experiments, each with 4 to 6 EDMs per group. Note that no significant difference was observed (nonparametric Kruskal wallis test followed by post hoc Dunn's multiple comparison test).

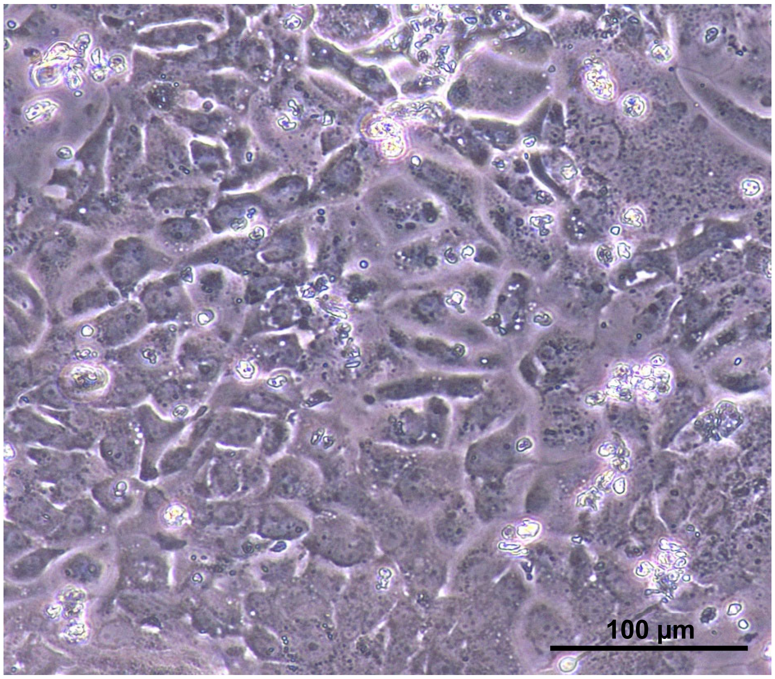

Figure S3. Representative bright-field image of EDM cultured for 5 days.
